# Investigation of the Effect of Hybrid Nanofiller on the Mechanical Performance and Surface Properties of Bio-Based Polylactic Acid/Polyolefin Elastomer (PLA/POE) Blend

**DOI:** 10.3390/polym15122708

**Published:** 2023-06-16

**Authors:** Nariman Rajabifar, Amir Rostami

**Affiliations:** 1 Department of Polymer Engineering and Color Technology, Amirkabir University of Technology (Tehran Polytechnic), Tehran P.O. Box 15875-4413, Iran; nariman.rf@aut.ac.ir; 2 Department of Chemical Engineering, Persian Gulf University, Bushehr P.O. Box 75169-13817, Iran

**Keywords:** polylactic acid, nanoclay, bio-based blend, rheology, surface analysis, mechanical performance

## Abstract

Polylactic acid has stood out among bio-based polymers for its usage in the food packaging industry and biomedical fields. Through the melt mixing process, the toughened poly(lactic) acid (PLA) was prepared with polyolefin elastomer (POE), incorporated via various ratios of nanoclay and a fixed amount of nanosilver particles (AgNPs). The correlation between the compatibility and morphology, mechanical properties, and surface roughness of samples with nanoclay was studied. The calculated surface tension and melt rheology confirmed the interfacial interaction demonstrated by droplet size, impact strength, and elongation at break. Each blend sample exhibited matrix-dispersed droplets, and the size of POE droplets steadily dropped with increasing nanoclay content, corresponding to the enhanced thermodynamic affinity between PLA and POE. Scanning electron microscopy (SEM) acknowledged that the inclusion of nanoclay in the PLA/POE blend ameliorated the mechanical performance by preferable localization in the interface of used components. The optimum value of elongation at break was acquired at about 32.44%, where the incorporation of 1 wt.% nanoclay led, respectively, to 171.4% and 24% enhancement rather than the PLA/POE blend with the composition of 80/20 and the virgin PLA. Similarly, the impact strength reached 3.46 ± 0.18 kJ m^−1^ as the highest obtained amount, showing the proximity of 23% progress to the unfilled PLA/POE blend. Surface analysis indicated that adding nanoclay caused the augment of surface roughness from 23.78 ± 5.80 µm in the unfilled PLA/POE blend to 57.65 ± 18.2 µm in PLA/POE contained 3 wt.% nanoclay. Rheological measurements implied that organoclay resulted in the strengthening of melt viscosity as well as the rheological parameters such as storage modulus and loss modulus. Han plot further showed that the storage modulus is always higher than the loss modulus in all prepared PLA/POE nanocomposite samples, corresponding to the restriction of polymer chains mobility induced by the formation of strong molecular interaction between nanofillers and polymer chains.

## 1. Introduction

Bio-based materials have lately gained significant attention from scientists to address environmental issues and reduce petrochemical materials dependence [1,2,3,4,5,6]. In the last few decades, poly(lactic) acid (PLA) has been deliberated as one of the most promising polymers among many bio-based engineering thermoplastics due to its high potential for substituting traditional polymers [7,8,9,10]. PLA is a biocompatible aliphatic polyester that could be easily commercialized from carbohydrate sources such as sugarcane, wheat, corn, and tapioca [11].

Despite advantages like high mechanical strength, transparency, slow degradation rate, and thermal processability compared to other biopolymers, which all make PLA an ideal candidate in biomedical fields and food packaging, some significant disadvantages restrict its usage [12,13]. In this regard, not only does PLA instinctively suffer from brittleness, hydrolysis, and low melt strength, but also the production of PLA is not that affordable compared to traditional polymers. To cover these shortages, several methods have been suggested such as adding nanofiller [14,15,16,17], plasticizing [18,19], copolymerizing with flexible monomers [20,21], and blending with tough materials [22].

There are several reports about compounding PLA with other polymers, including elastomers, copolymers, thermoplastics, and biodegradable polymers [23,24,25,26,27]. Due to their superior elasticity and impact-modifying role, polyolefin elastomers (POEs) have newly stood out in academia [28,29,30,31]. This synthesized thermoplastic elastomer (TPE) also features anti-aging properties, enhancing the melt strength of polymeric blends, excellent processability, and favorable thermal stability.

In this respect, Zhou et al. [32] reported an improvement in the impact strength of PLA blend toughened by POE, approaching an optimum value by incorporation of 15 wt.% POE (~12.5 kJ m^−2^). The tensile strength also showed a constant downward trend by adding POE to PLA from ~63 MPa to 28 MPa with 20 wt.% POE. Daneshpayeh et al. [33] investigated the mechanical properties of PLA/POE blend in ratios of 90/10 and 80/20, containing different proportions of multi-walled carbon nanotubes (MWCNTs) and graphene nanoplatelets (GnPs). It was found that blending PLA with 20 wt.% POE resulted in a better elongation at break (~35%) than blending it with 10 wt.% POE (~32%). Furthermore, it was reported that PLA/POE blends deformed poorly when both MWCNTs and GnPs were added separately and simultaneously. However, it was seen through the addition of 1 wt.% GnPs to the PLA/POE matrix that deformation at break increased by 9%. When it comes to impact strength test, PLA/POE with the compositions of 90/10 and 80/20 showed nearly 8.5 J m^−1^ and 12 J m^−1^ values, respectively, compared to 5.21 J m^−1^ of neat PLA. By adding either MWCNTs or GnPs, the impact strength values were continuously decreased because of the rigidity of nanofillers, leading to the brittle samples. In addition, the influence of a constant amount of polyolefin elastomer-graft-maleic anhydride (POE-g-MA) as a compatibilizer accompanied by different ratios of halloysite nanotube (HNT) on the PLA/POE blend was studied by Jalalifar et al. [34]. It was claimed that the size of dispersed POE droplets in the PLA matrix was diminished by introducing POE-g-MA because of the mechanical interaction between POE and POE-g-MA, considering the advancement of thermodynamic affinity of components.

In the past few years, organic-inorganic hybrids have drawn considerable attention due to their synergistic properties generated by the two components combined. There are many reports revealing that polymer/nanoclay nanocomposites perform better mechanically than their unloaded counterparts with only small amounts of nanoclay [35,36,37,38]. One example is the effect of nanoclay Cloisite 30B contents on tensile and impact properties of melt-mixed PLA, reported by Asadi et al. [39], where an enhancement in elongation at break seen by the inclusion of 2 and 3 wt.% nanoclay. It was found that the sample containing 3 wt.% of Cloisite 30B had the highest tensile strength and elongation at break, and featured more flexibility and energy absorption ability compared to the neat PLA. Moreover, a reduction trend of about 15% was observed in the impact strength of virgin PLA (32 J m^−1^) in PLA/nanoclay samples using Cloisite 30B.

In this work, an attempt was made to look into the impact of different nanoclay content on the mechanical performance, surface properties, and development of the microstructure of PLA/POE nanocomposites fabricated by the melt mixing method. We also constantly contained 1 wt.% AgNPs to compensate the loss of Young’s modulus when POE was added to the virgin PLA. As part of this study, structural characterization was conducted using scanning electron microscopy (SEM) and atomic force microscopy (AFM). A Rheometrics mechanical spectrometer (RMS) was also employed to study the viscoelastic behavior of samples, as well as a tensile machine and an impact tester to assess their mechanical performance.

## 2. Experimental

### 2.1. Materials

Ingeo^®^ biopolymer PLA grade 2003D from NatureWorks Co. (Plymouth, MN, USA) was used in this study with a density of 1.24 g cm^−1^, melt flow index of 0.6 g min^−1^ (2.16 kg at 210 °C), and L/D isomeric ratio = 96/4. POE grade DF640 was purchased from Mitsui Chemical (Tokyo, Japan), having a density of 0.86 g cm^−1^, melt flow index of 0.36 g min^−1^ (2.16 kg at 190 °C), and shore 56 A. Organoclay (Cloisite 30B) as natural montmorillonite modified with a quaternary ammonium ion containing methyl tallow bis-2-hydroxyethyl was provided from BYK Additives and Instruments (Wesel, Germany) with d-spacing of 18.5 Å and a bulk density of 0.228 g cm^−1^. Silver nanoparticles (AgNPs) with particle size < 20 nm produced by US Nano (Houston, TX, USA) were also used in this work.

### 2.2. Sample Preparation and Coding

Virgin PLA and PLA/POE nanocomposites were fabricated via the melt compounding method using a laboratory-size internal mixer (Brabender, Duisburg, Germany) under the temperature of 180 °C and 60 rpm rotor speed. To eliminate the influence of moisture on the results, all materials were dehumidified in a vacuum oven at 50 °C for 10 h prior to preparation. In order to fabricate nanocomposites, PLA and POE were blended for 3 min until a uniform melt fluid was obtained, then Cloisite 30B and AgNPs were simultaneously added. The melting process endured for 7 min. All extracted compounds were molded into the 1 mm thick sheet between two plates of the hydraulic hot press (400S, Polystat model, Offenburg, Germany) for 5 min pre-heat and 1 min under molding pressure of 50 MPa with a temperature of 190 °C. Subsequently, samples were cooled at the same pressure for 10 min at room temperature. Table 1 illustrates the composition and coding of the prepared compounds.

### 2.3. Morphology Characterization

To investigate the developed microstructure and dispersion state of nanomaterials, fractured surfaces were observed using a Quanta 200 ESEM (FEI Company, Hillsboro, OR, USA) instrument at an acceleration voltage of 30 kV, equipped with an energy dispersive X-ray spectroscopy (EDX) EDS silicon drift detector to capture an elemental map of the atomic elements present in the nanocomposite samples. Each sample was cryogenically fractured after immersion in liquid nitrogen for 15 min. All specimens were sputtered with a thin layer of gold to diminish electron beam damage prior to microscopic observation and Map-EDX analysis. Furthermore, atomic force microscopy (AFM) was carried out to investigate the size and 3D structure of nanomaterials, using Universal SPM (Ambios Technology, Santa Cruz, CA, USA). AFM was performed under ambient conditions in nondestructive mode (tapping) with a standard silicon cantilever probe tip on the thin film samples. Topographic and phase images were recorded simultaneously.

### 2.4. Rheological Characterization

Since the melt viscoelastic behavior of the samples has a crucial effect in directing the morphology of the blend, rheological analysis was studied using a rheometric mechanical spectrometry (RMS), MCR 302 rheometer (Anton Paar, Graz, Austria) at 180 °C equipped with parallel plate configuration (diameter = 25 mm, constant gap = 1 mm). Before rheological characterization, specimens were compression molded into 1 mm thickness and 25 mm diameter. Frequency sweep was obtained for each sample, noting that all measurements were performed under the atmosphere of dry nitrogen to hinder oxidative degradation. The oscillatory shear test was carried out in a range of 0.01–600 rad s^−1^ at strain amplitude set at 1% to maintain the response of blends within the linear viscoelastic regime.

### 2.5. Mechanical Properties Measurement

The mechanical properties were measured by Galdabini-Sun 2500 (Galdabini, Cardano Al Campo, VA, Italy) instrument at a rate of 500 mm min^−1^ based on ASTM D-638 standard at room temperature. The notched Izod impact test was performed using an impact tester (Ueshima Seisakusho Co., Tokyo, Japan) in accordance with ASTM-D256. The mechanical properties results were averaged over five replicates of each sample.

## 3. Results and Discussion

### 3.1. Morphology Development in PLA/POE Blends Containing Nanoclay

**Figure 1:**
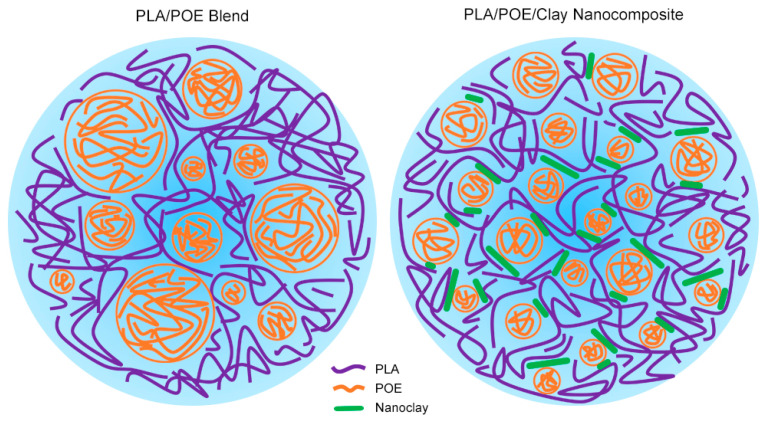
Schematic of PLA/POE blend and PLA/POE/clay nanocomposite. This figure shows the size of POE droplets declined with the incorporation of Cloisite 30B regarding its localization in the interface.

Morphology development is considered a central parameter for the different ultimate properties of samples in polymeric blends, for instance, mechanical performance and permeability to gas. In order to have an insight on microstructure revolution in prepared samples, SEM analysis was individually conducted on cryogenically fractured surface of blends. As can be seen in Figure 1, the mixture of PLA and POE with the composition of 80/20 represents a droplet-matrix morphology, where the POE phase is distributed within the PLA matrix. This microstructure could be corresponded to (1) POE having a higher viscosity than the virgin PLA phase and usual use as an impact modifier or with the intention of increasing the toughness, and (2) blending of PLA and POE alongside the noted proportion, due to the different surface free energy of PLA and POE given in Table 2, could lead to the formation of phase-dispersed morphology [40]. Regardless of experimental observations, the formation of this microstructure could also be predicted theoretically considering the viscosity and volume fraction of each component, as Miles and Zurek suggested [41].

By introducing nanoparticles in polymeric blends, the final properties correlated to the microstructure of samples would be influenced by their localiztion [45]. Accordingly, surface energy and interfacial tension are important in terms of the selective implantation of nanoparticles [46]. Using the geometric-mean equation, the interfacial tension between the components in the blend, i and j, were calculated:

(1) $$\gamma_{ij}=\gamma_{i}+\gamma_{j}-2\;(\sqrt{\gamma_{i}^{d}\gamma_{j}^{d}}+\sqrt{\gamma_{i}^{p}\gamma_{j}^{p}})$$

where $\gamma_{ij}$ represents the interfacial tension between blend components (i,j), and $\gamma_{i}$ and $\gamma_{j}$ are the total surface tension of components. $\gamma_{i}^{p}$ and $\gamma_{i}^{d}$ are the polar and dispersive surface tensions of component i. Concerning the Young’s relation, it is possible to predict the distribution of nanoparticle within a polymer blend by the wetting coefficient ($\omega_{a}$) with droplet-matrix morphology as follows:

(2) $$\omega_{a}=\frac{\gamma_{Clay\text{-}POE}-\gamma_{Clay\text{-}PLA}}{\gamma_{PLA\text{-}POE}}$$

where $\gamma_{Clay\text{-}POE}$ and $\gamma_{Clay\text{-}PLA}$ are subsequently the interfacial tensions between the nanoclay and POE as dispersed phase and PLA matrix, whereas $\gamma_{PLA\text{-}POE}$ is the interfacial tension between the PLA and POE. As a result, using the mentioned equations and calculating $\omega_{a}$, we can estimate the localization of nanoclay in the binary blend of PLA and POE. The nanoclay particles are located within the POE phase if $\omega_{a}$ > 1, they are located within the PLA phase if $\omega_{a}$ < −1, and eventually the nanoclay particles disperse at the interface if −1 < $\omega_{a}$ < 1.

Regarding the wetting coefficient figure, $\omega_{a}=-0.86$, it is predicted that nanoclay particles tend to be dispersed in the interface as shown in Figure 1. Table 3 illustrates the interfacial tension values calculated by the above-mentioned equations.

**Figure 2:**
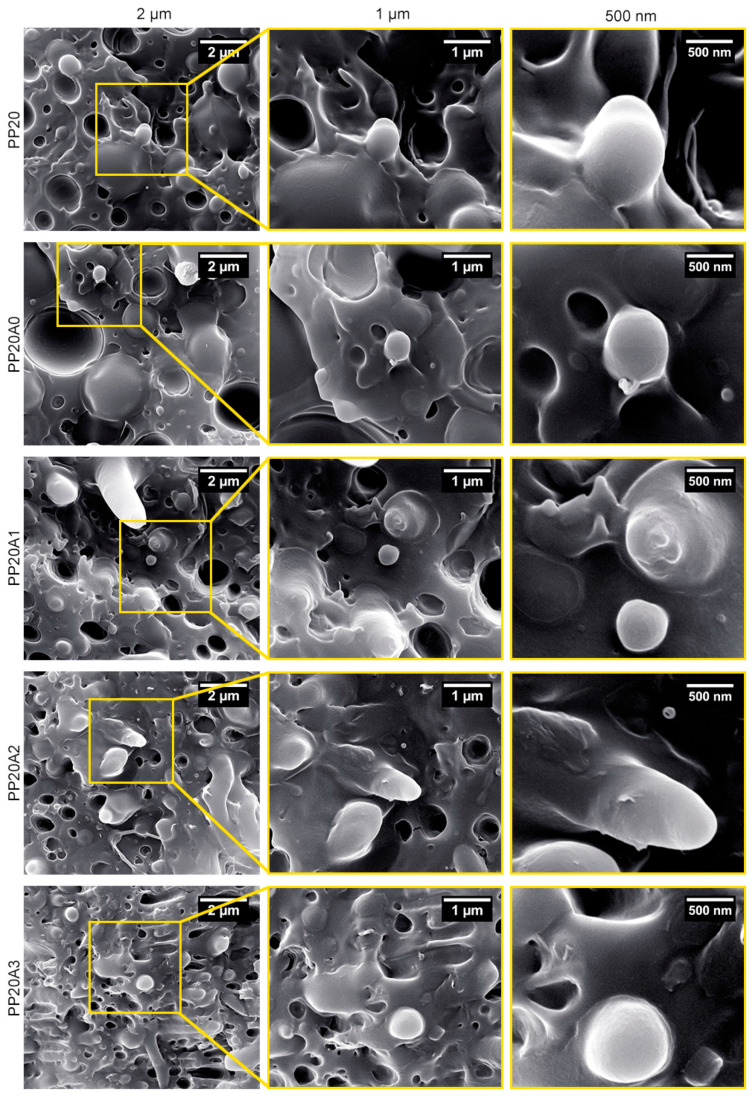
SEM micrographs of prepared PLA/POE blend and its nanocomposites containing 1 wt.% AgNPs and 0–3 wt.% nanoclay. Scale bar: 2 µm, 1 µm, and 500 nm.

By the overlook at SEM micrographs of nanocomposites in Figure 2, it could be understood that the particle size of the dispersed phase (POE) was efficiently diminished due to the presence of organoclay. In fact, as the estimated wetting coefficient showed, localization of nanoclay brought about the reduction in POE droplet coalescence at the interface, which can be justified by the improvement of thermodynamic affinity between used components and the compatibilizer role of Cloisite 30B. Regardless of theoretical equations, Figure 2 displays that not only does nanoclay tend to be dispersed into the interface between PLA and POE, but it also indicates that the presence of nanoclay in the noted sphere has prompted the declining of POE droplet sizes. Further, it has been suggested that incorporating organoclay into a multiphase polymeric blend could possibly decrease the coalescence of polymer droplets of the minor phase, corresponding to the presence of a solid barrier [44]. Therefore, per previous reports [47], minimizing droplet size in phase-separated morphology could be perceived as superior toughness conversely to the virgin PLA.

**Figure 3:**
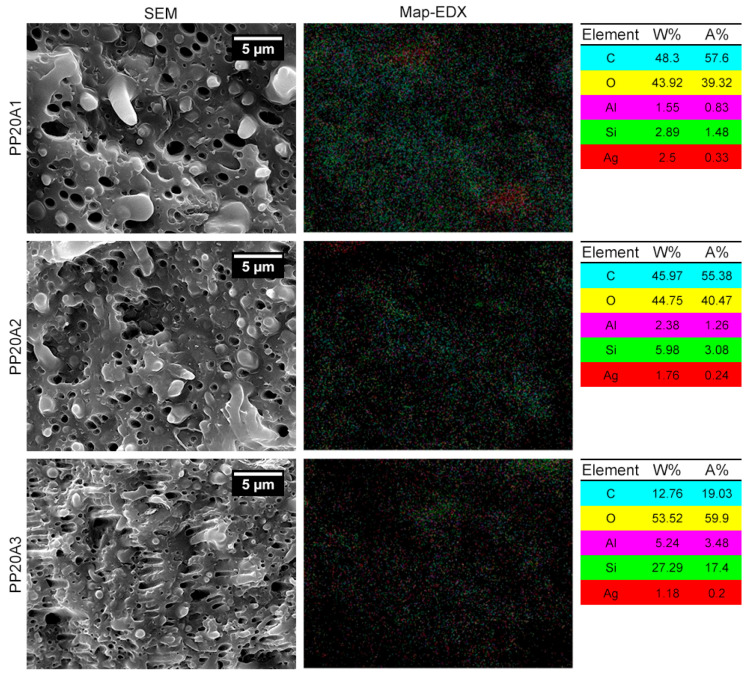
SEM micrographs and Map-EDX with elemental analysis of PLA/POE blend and PLA/POE nanocomposites containing 1 wt.% AgNPs and 0–3 wt.% nanoclay. Scale bar: 5 µm.

Figure 3 illustrates the mapping of the samples using energy dispersive X-ray spectroscopy (EDX) to detect the elemental distribution of nano particles through the blends. As can be seen, nanoclay was homogenously dispersed in all the samples according to the fact that the number of green spots labeled silicon (Si), as one key element in organoclays, constantly increased by enhancing the amount of nanoclay. Nonetheless, some agglomeration of nanoclay particles was observed in the prepared samples, particularly in the PP20A3, which has the maximum loading of Cloisite 30B content (3 wt.%). Moreover, the proportion of AgNPs, respectively, fell in the PP20A1, PP20A2, and PP20A3 samples, which can be correlated to the increment of nanoclay content. Although the dispersion and distribution of AgNPs in the PP20A2 and PP20A3 seems fine, there are two areas of aggregating nanosilver particles shown with red points in PP20A1.

### 3.2. Surface Analysis of PLA/POE Blend and PLA/POE Nanocomposite Samples

**Figure 4:**
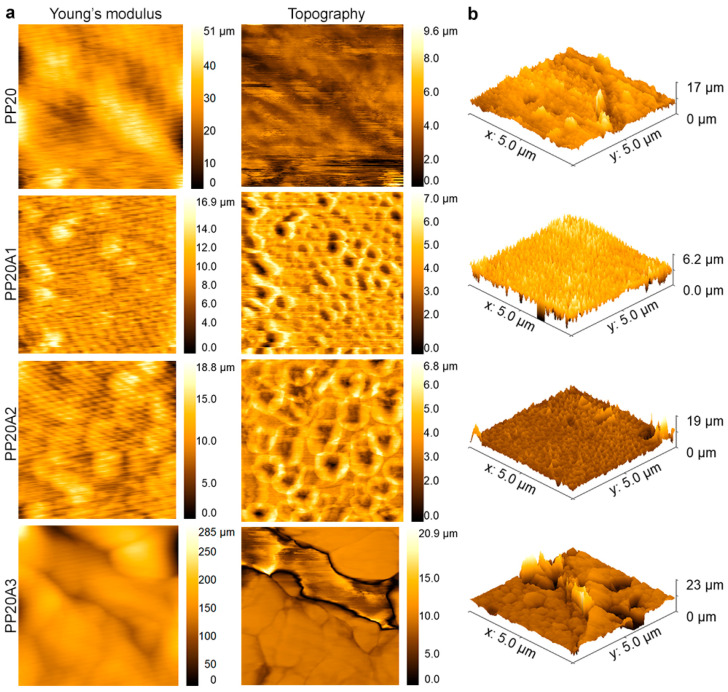
AFM images of PLA/POE blend and PLA/POE nanocomposites in two- and three-dimensional images: (**a**) Young’s modulus and topography images, (**b**) three-dimensional micrographs. Scale bar: (**a**) 2 µm, (**b**) 5 µm.

Figure 4 depicts the AFM two- and three-dimensional surface images of the PLA/POE blends and the PLA/POE nanocomposite compositions with nanoclay amounts, ranging from 1 to 3 wt.%. In order to indicate an insight into the effect of nanoclay on the surface morphology and topography on the samples, tapping mode as a nondestructive method was employed to observe the surface properties of PLA/POE blends. On the phase micrograph, the dark-colored phase corresponds to the phase with a lower modulus, which is POE, whereas the light shade corresponds to the PLA phase as the matrix and/or nanoparticles. Figure 4a illustrates a fine dispersed POE phase through the PLA without nanoparticles, although some accumulated POE content can be seen. In the PP20 sample, the bright areas conform to the PLA component with a higher modulus rather than POE.

According to what was formerly explained in SEM micrographs, the droplet-matrix morphology of prepared blends was seen in the PP20A1 and PP20A2 topography images even though AFM is fundamentally a surface analysis technique. This means the mentioned morphology could have appeared on the samples’ surface, confirming the electron microscopy images. Figure 4a indicates that adding 1 wt.% nanoclay increases surface modulus of the PP20A1 in comparison to the PP20, regarding the light shades. This conforms to the previous studies on the effect of nanoclay on the surface properties of PLA [48]. Moreover, 2 wt.% nanoclay has caused a better dispersion of POE in the matrix if Young’s modulus image of the PP20A2 equates to the PP20. Yet, further content of Cloisite 30B has led to observing the accumulation of POE, which could be related to the agglomeration of nanoclay and weak interaction between polymer chains and nanoparticles.

Looking at the provided 3D images of samples in 5 µm scale, it is obvious that adding nanoclay has broadly brought their surfaces about rougher. This acknowledges that the presence of nanoclay could not only affect the microstructure of the polymer system but also the surface of the sample [39]. Although the incorporation of 1 wt.% nanoclay decreased the average surface roughness ($R_{a}$) of the PLA/POE sample as it has illustrated in Table 4, the measured $R_{a}$ showed a dramatic increment from 10.41 ± 9.44 µm to 57.65 ± 18.18 µm in surface roughness, as well as roughly 17 µm to 19 and 23 µm in height average by adding 2 and 3 wt.% nanoclay, respectively. This demonstrates that 1 wt.% loading of Cloisite 30B had a greater influence on the sample, at least on the surface, to reduce formed POE droplets compared to the PP20A2.

### 3.3. Rheological Properties of PLA/POE Nanocomposites

**Figure 5:**
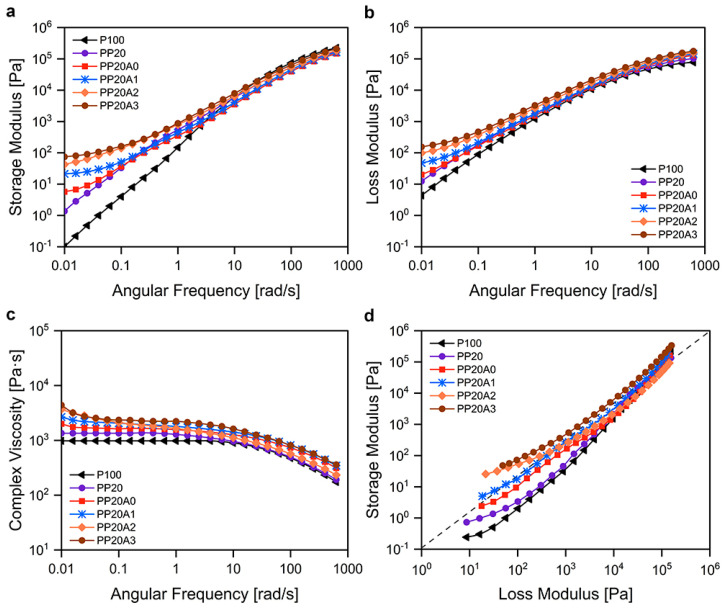
Rheological behavior of PLA/POE samples as a function of angular frequency: (**a**) storage modulus, (**b**) loss modulus, and (**c**) complex viscosity. (**d**) Han plots of prepared samples.

The rheological study of nanocomposites presents a worthwhile insight into the microphase structure, interfacial actions, and the architecture of polymeric chains. Thereby analyzing the low-frequency dynamic response of the material as an indirect measure of chain mobility, the exfoliation state of a nanocomposite containing nanoclay can be investigated. Along with this perspective, the study of the viscoelastic behavior of polymer blends with different compositions is important when it comes to their processability. Figure 5 indicates the rheological behavior of prepared virgin PLA, PLA/POE blend, and PLA/POE nanocomposites. Showing the variation of storage modulus (G′) and loss modulus (G″) during frequency sweep (*w*) in Figure 5a,b, it can be seen that the behavior of virgin PLA (code: P100) is liquid-like. In the low frequency region, the slope of G′ and G″ of the P100 are subsequently ~2 and 1, corresponding to the power-law rheology (G′~*w*^2^ and G″~*w*). What is more, at low frequencies, it is evident that the value of storage and loss modulus of the PP20 are higher than the P100. It has been suggested by Shin et al. that this behavior lies in the shape relaxation of dispersed phase (POE) into the continuous phase, which is PLA [40].

In addition, a solid-like response appeared for PLA/POE nanocomposites at initial frequencies, which may well result from an organoclay network formed into the blend. Indeed, as a result of restricted polymer chain mobility caused by firm molecular interactions with nanofillers, the deviation of storage modulus often takes place. This behavior is where G′ reaches a plateau in initial frequencies, so-called nonterminal. Accordingly, the storage modulus of nanocomposites increased alongside feeding increased proportion of nanoclay into the binary system, regarding the given curves in Figure 5a. The equivalent trend can be seen for the loss modulus curves. Nanoclay could therefore be viewed as a reinforcing component in PLA and PLA/POE binary blends, leading to an increase in melt modulus.

Looking at the given complex viscosity (η*) of prepared samples over the range of low and high angular frequencies in Figure 5b, it is manifested that the PP20 has a higher viscosity than the virgin PLA (code: P100), and both samples exhibit Newtonian behavior, as well as weak shear-thinning. The superior η* value of the PP20 is because of the higher viscosity of POE component indeed. By introducing nanosilver particles and then nanoclay to the binary PLA/POE blend, the figure of η* samples tends to increase and exhibit a complex viscosity upturn, furthering with loading more nanoclay component. As a result, polymer chains may be unable to move segmentally due to a network-like microstructure leading them to be restricted.

The Han plot (G′ vs. G″) of prepared samples was displayed in Figure 5d so as to evaluate the difference between the structure of the virgin PLA, its blend, and PLA/POE nanocomposites. This curve signifies the change from liquid-like to solid-like viscoelasticity where G′ = G″. In the case of the P100 and the PP20 mixture, the values of G″ constantly exceed G′ which reveals the liquid-like behavior and viscous nature. The PP20A2 curve at the low frequency of Han plot, however, shows a crossing point between storage and loss modulus. Upon the formation of nanolayer networks in the polymeric media, the viscoelastic behavior of this nanocomposite changes from a liquid-like to a solid-like state.

### 3.4. Mechanical Performance of the Prepared PLA/POE Nanocomposites

**Figure 6:**
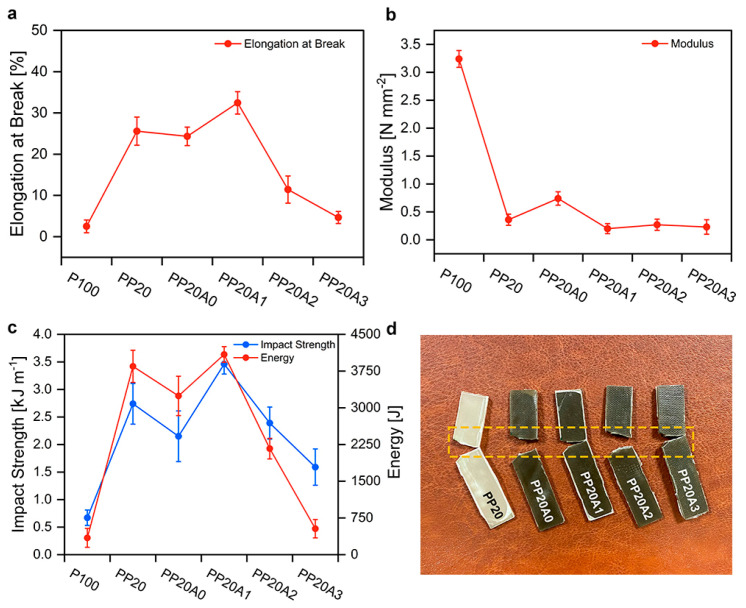
Mechanical properties of PLA/POE nanocomposites: (**a**) elongation at break, (**b**) impact strength, (**c**) modulus and energy, (**d**) picture of fractured samples after performed Izod test.

Using five repeated tests for each sample, the average value of the mechanical properties of the samples has been illustrated in Figure 6. As expected, the minimum amount of elongation at break earmarks to the virgin PLA (code: P100) at 2.5 ± 1.55% due to its instinct brittleness. Figure 6a states that the elongation at break of the P100 was significantly enhanced by introducing 20 phr of POE (code: PP20), approaching 25.59 ± 3.41%. This ductile behavior is attributed to the flexibility of POE as a toughened thermoplastic [49].

Figure 6b,c show that utilizing the toughened component, compared to the P100, has brought about the figure of modulus and Izod impact strength to a major change with 160% and 121.4%, respectively. As can be seen in the PP20A0 sample which contains 1 phr of nanosilver particle, the elongation at break and impact strength slightly declined in comparison to the preceding sample. This could be associated with the high modulus of nanosilver which caused not only a superior modulus among blended and nanocomposite samples, but also confined the mobility of polymer chains, leading to the lower energy absorption of Izod test (Figure 6b) rather than the PP20 [50].

Based on Figure 6, the optimum mechanical properties of PLA/POE nanocomposite were observed in the PP20A1, where the proportion of 1 phr noticeably increased the toughness of the nanocomposites when evaluated in terms of increasing the elongation at break and impact strength for the PLA/POE nanocomposite. In particular, the elongation at break and Izod impact values correspondingly reached 32.44 ± 2.71% and 3.46 ± 0.18 kJ m^−1^, which implies approximately 24% and 23% enhancement relative to the PP20. Moreover, Figure 6b indicates the maximum energy of tensile measurements throughout the prepared composites, standing at 4089.58 ± 159.66 J. Nevertheless, loading more content nanoclay caused the reduction in mechanical performance. A downward trend in elongation at break of the PP20A2 and PP20A3, 11.43 ± 3.31% and 4.65 ± 1.46%, was subsequently observed even though their toughness is still more remarkable than the virgin PLA.

The same alteration was obtained for the Izod test, where the PP20A2 showed 2.39 ± 0.29 kJ m^−1^ of impact strength, followed by a significant decrease to 1.59 ± 0.33 kJ m^−1^. This behavior is interpreted by unfavorable dispersion and distribution of nanoclay in the PLA/POE nanocomposites. In other words, as previously explained by rheology measurements, 2 and 3 phr loading of nanoclay led to the occurrence of agglomerations. The quantitative value of impact strength and tensile properties are presented in Table 5.

## 4. Conclusions

The PLA/POE blends were prepared via the melt mixing method as well as their nanocomposites containing different ratios of nanoclay and their effect on the mechanical performance, surface properties, microstructure development, and rheology measurements were investigated. In all samples, the dispersed phase morphology was observed. By micrographs of microscopy techniques, it was proven that the presence of nanoclay led to a decline in the size of formed POE droplets in the PLA matrix on account of the interaction and affinity improvement between used components. This affected the tensile properties and impact strength of the samples to reach higher values compared to the virgin PLA and PLA/POE blend without nanofillers. PLA/POE blend incorporated 1 wt.% nanoclay, respectively, showed the optimum elongation at break and impact strength, 32.44 ± 2.71% and 3.46 ± 0.18 kJ m^−1^, indicating the efficiency of the low ratio of nanoclay on the toughening. These figures were significantly higher than the virgin PLA with only 2.5 ± 1.55 elongation at break and 0.67 ± 0.14 kJ m^−1^ impact resistance. The study of the surface properties revealed that PLA/POE nanocomposites exhibited rougher surfaces compared to the neat PLA/POE blend. The estimated roughness showed the sample containing 3 wt.% nanoclay featured nearly 83% more than the unfilled PLA/POE sample. As a result of the rheological analysis of the blends, we found that PLA and POE were more compatible by adding Cloisite 30B, which was in agreement with the data on the interfacial tension and the morphology of the blend.

## Figures and Tables

**Table 1:** Composition and coding of prepared samples in this study.

Sample	Sample Coding	AgNPs [wt.%]	Component Composition		
			PLA	POE	Organoclay
Virgin PLA	P100	-	100	-	-
PLA/POE	PP20	-	80	20	-
PLA/POE/AgNPs	PP20A0	1	80	20	0
PLA/POE/AgNPs/Organoclay	PP20A1	1	80	20	1
	PP20A2	1	80	20	2
	PP20A3	1	80	20	3

**Table 2:** The calculated surface free energy of components.

Material	*γ* (mN m^−1^)	*γ^p^* (mN m^−1^)	*γ^d^* (mN m^−1^)	Reference
PLA	33.9	3.9	30	[42]
POE	17.45	1.89	15.56	[43]
Cloisite 30B	35	12.6	22.4	[44]

**Table 3:** Calculated interfacial tension for sample pairs.

Sample Pairs	Interfacial Tension (mN m^−1^)	Code
PLA-POE	2.71	$\gamma_{PLA\text{-}POE}$
Clay-PLA	3.03	$\gamma_{Clay\text{-}PLA}$
Clay-POE	5.35	$\gamma_{Clay\text{-}POE}$

**Table 4:** Calculated surface roughness and height average of prepared samples.

Sample	$R_{a}$ [µm]	Height Average [µm]
PP20	23.78 ± 5.80	17.08 ± 3.56
PP20A1	8.57 ± 1.39	6.24 ± 2.19
PP20A2	10.41 ± 9.44	19.01 ± 2.38
PP20A3	57.65 ± 18.18	23.04 ± 9.04

**Table 5:** Tensile properties and impact strength of neat PLA, PLA/POE blend, and PLA/POE nanocomposites.

Sample	Tensile Strength [N mm^−2^]	Modulus [N mm^−2^]	Elongation at Break [%]	Energy [J]	Impact Strength [kJ m^−1^]
P100	60.03 ± 15.06	3.24 ± 0.15	2.50 ± 1.55	343 ± 192.21	0.67 ± 0.14
PP20	37.80 ± 9.51	0.36 ± 0.18	25.59 ± 3.41	3846.51 ± 328.65	2.74 ± 0.37
PP20A0	36.06 ± 11.48	0.74 ± 0.12	24.33 ± 2.24	3243.86 ± 401.92	2.15 ± 0.46
PP20A1	28.09 ± 3.35	0.20 ± 0.09	32.44 ± 2.71	4089.58 ± 159.66	3.46 ± 0.18
PP20A2	33.76 ± 6.19	0.27 ± 0.11	11.43 ± 3.31	2166.43 ± 210.78	2.39 ± 0.29
PP20A3	29.00 ± 10.03	0.23 ± 0.13	4.65 ± 1.46	530.49 ± 188.44	1.59 ± 0.33

## Data Availability

The data that support the findings of this study are available from the corresponding author upon reasonable request.
